# Recent advances in the treatment of chronic heart failure

**DOI:** 10.12688/f1000research.20447.1

**Published:** 2019-12-20

**Authors:** Leo F Buckley, Amil M Shah

**Affiliations:** 1 Department of Pharmacy, Brigham and Women's Hospital, Boston, USA; 2 Division of Cardiovascular Medicine, Brigham and Women’s Hospital, Boston, USA

**Keywords:** heart failure, therapy

## Abstract

After more than a decade of relatively modest advancements, heart failure therapeutic development has accelerated, with the PARADIGM-HF trial and the SHIFT trial demonstrated significant reductions in cardiovascular death and heart failure hospitalization for sacubitril-valsartan and in heart failure hospitalization alone for ivabradine. Several heart failure therapies have since received or stand on the verge of market approval and promise substantive advances in the treatment of chronic heart failure. Some of these improve clinical outcomes, whereas others improve functional or patient-reported outcomes. In light of these rapid advances in the care of adults living with chronic heart failure, in this review we seek to update the general practitioner on novel heart failure therapies. Specifically, we will review recent data on the implementation of sacubitril-valsartan, treatment of functional mitral regurgitation, sodium-glucose co-transporter-2 (SGLT-2) inhibitor therapy, agents for transthyretin amyloid cardiomyopathy, treatment of iron deficiency in heart failure, and the use of biomarkers or remote hemodynamic monitoring to guide heart failure therapy.

## Introduction

After a decade without successful development of novel heart failure therapeutics, the PARADIGM-HF trial and the SHIFT trial demonstrated significant reductions in cardiovascular death and heart failure hospitalization for sacubitril-valsartan and in heart failure hospitalization alone for ivabradine. Since the market introduction of these two therapies, several heart failure therapies have received or stand on the verge of market approval. In light of the rapid advances in the care of adults living with chronic heart failure, we have sought to update the general practitioner on heart failure therapies approved during this wave of successful drug development.

## Implementation of sacubitril-valsartan

Two clinical trials have tested novel strategies for the safe and effective implementation of sacubitril-valsartan in 498 patients with heart failure and a reduced ejection fraction. The TITRATION study compared the safety and efficacy of conservative and condensed up-titration schedules for sacubitril-valsartan in ambulatory patients ^1^. All patients received sacubitril-valsartan 24/26 mg twice daily for 5 days during an open-label, run-in period followed by randomization and two additional treatment periods. At randomization, patients in the conservative arm continued sacubitril-valsartan at 24/26 mg twice daily whereas patients in the condensed arm were titrated to 49/51 mg twice daily. After 2 weeks, the conservative arm increased to 49/51 mg twice daily and the condensed arm increased to the maximal dose of 97/103 mg twice daily. The condensed arm then continued the maximal 97/103 mg twice daily for the remaining 9 weeks of the study, whereas the conservative arm continued the 49/51 mg twice-daily regimen for 3 weeks before increasing to the maximal 97/103 mg twice daily for 6 weeks. In aggregate, the condensed arm reached the maximal dose with one fewer visit than the conservative arm and increased from 24/26 mg to 49/51 mg 2 weeks earlier than the conservative arm and from 49/51 mg to 97/103 mg 3 weeks earlier than the conservative arm.

The proportion of patients experiencing any hypotension (9.7% vs. 8.4%; *P* = 0.57) and systolic blood pressure less than 95 mm Hg (8.9% vs. 5.2%; *P* = 0.10) was not significantly different between the conservative and condensed arms. Serum potassium of more than 5.5 mmol/L occurred in 7.3% of condensed and 4% of conservative patients ( *P* = 0.10). There was no significant difference in renal dysfunction between the two arms (7.3% vs. 7.6%; *P* = 0.99). Adverse effects occurred most frequently in patients who switched to sacubitril-valsartan from a low dose of an angiotensin-converting enzyme inhibitor (≤10 mg of enalapril or equivalent) or angiotensin receptor blocker (≤160 mg of valsartan or equivalent) and those who had baseline systolic blood pressure of 100 to 110 mm Hg. Less than one fifth of patients with heart failure reach target doses of renin–angiotensin–aldosterone inhibitors.

The randomized, double-blind PIONEER-HF trial compared in-hospital initiation of sacubitril-valsartan to initiation or enalapril among 881 patients with stabilized decompensated heart failure ^2^. Change in N-terminal pro B-type natriuretic peptide (NT-proBNP) from baseline to week 8 was significantly greater in the sacubitril-valsartan group than the enalapril group (geometric mean ratio vs. baseline: 0.53 for sacubitril-valsartan vs. 0.75 for enalapril; between-group percent change: −47% vs. −25%; *P* <0.001). Sacubitril-valsartan had no effect on a secondary 7-point composite clinical endpoint but was associated with a reduced risk of rehospitalization for heart failure (35 [8%] vs. 61 [14%]; hazard ratio [HR] 0.56, 95% confidence interval [CI] 0.37 to 0.84; *P* = not reported). Of note, the trial protocol aimed to achieve maximal sacubitril-valsartan doses within 1 week if tolerated. Slightly more patients experienced worsening renal function (13.6% vs. 14.7%), hyperkalemia (11.6% vs. 9.3%), and symptomatic hypotension (15.0% vs. 12.7%) but these differences did not reach statistical significance.

**Figure 1.**
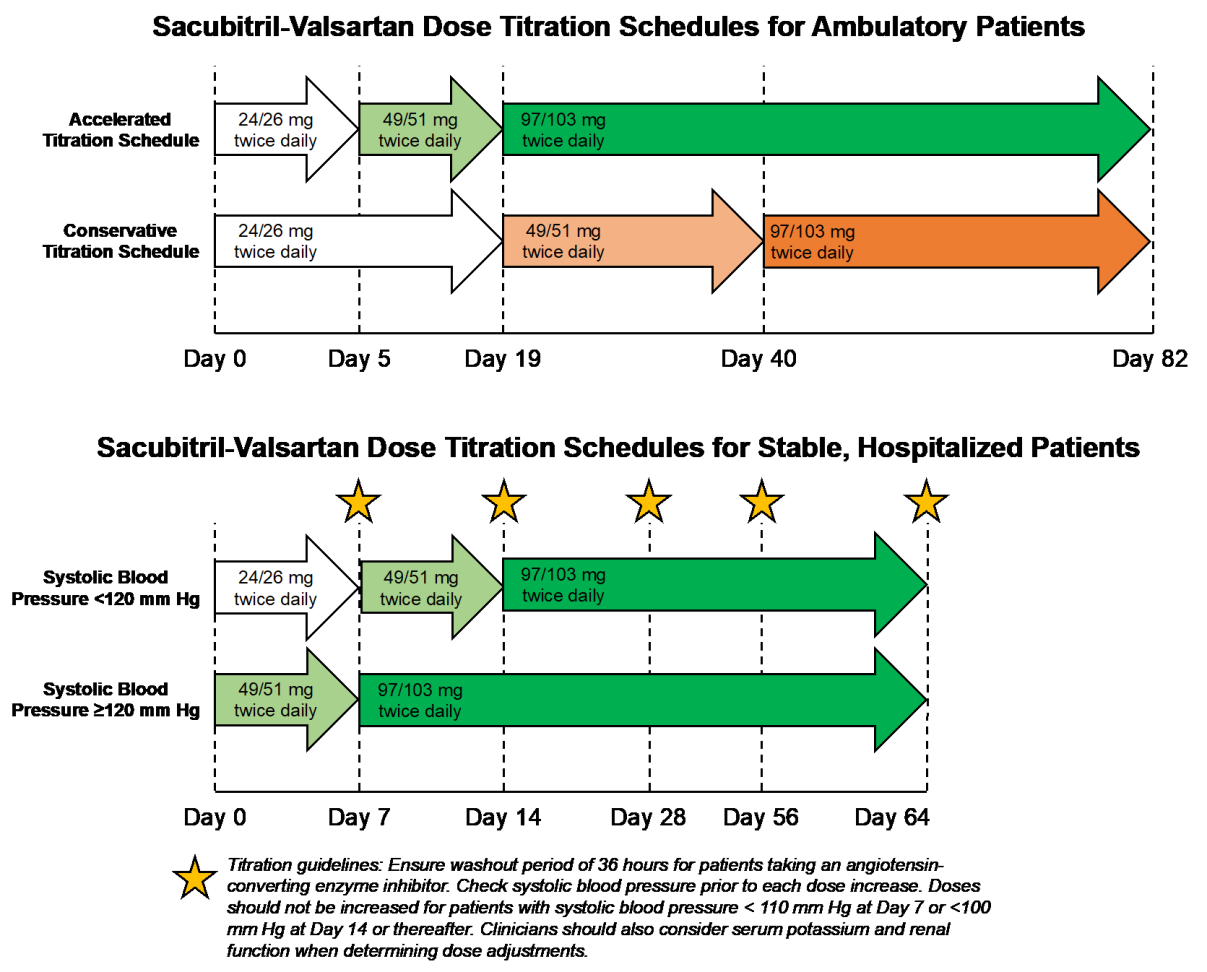
Titration schedules for ambulatory and hospitalized patients initiating sacubitril-valsartan.

These important trials provide clinicians with structured protocols ( Figure 1) to maximize sacubitril-valsartan doses in hemodynamically stable, normokalemic patients with heart failure and a reduced ejection fraction and relatively intact renal function (estimated glomerular filtration rate of at least 30 mL/min per 1.73 m ^2^).

## Sacubitril-valsartan for the treatment of heart failure with preserved ejection fraction

The PARAGON-HF trial tested the hypothesis that sacubitril-valsartan lowers the rate of a composite outcome of total heart failure hospitalizations and cardiovascular death compared with valsartan alone in patients with heart failure and preserved ejection fraction ^3^. In addition to a left ventricular ejection fraction (LVEF) of at least 45%, patients were required to have additional objective criteria of heart failure, including an elevated natriuretic peptide level, structural heart disease (left atrial enlargement or increased left ventricular wall thickness), and diuretic use. Patients were ineligible if they had a previous LVEF of less than 40%. After a single-blind run-in phase, 4796 patients were randomly assigned to sacubitril-valsartan (target dose of 97/103 mg twice daily) or valsartan (target dose of 160 mg twice daily) and followed for a median of 35 (interquartile range of 30 to 41) months.

The number of composite heart failure hospitalizations or cardiovascular deaths was nominally lower in the sacubitril-valsartan arm than in the valsartan arm (526 vs. 1009; rate ratio 0.87, 95% CI 0.75 to 1.01; *P* = 0.06) but did not achieve statistical significance. Randomization to sacubitril-valsartan was associated with a smaller total number of heart failure hospitalizations (690 vs. 797; rate ratio 0.85, 95% CI 0.72 to 1.00) but no difference in death due to cardiovascular causes (204 [8.5%] vs. 212 [8.9%] in the sacubitril-valsartan and valsartan arms respectively) or due to any cause (342 [14.1%] vs. 349 [14.6%]). Although New York Heart Association functional class remains unchanged at 8 months in most patients, more sacubitril-valsartan patients than valsartan patients appeared to have investigator-assessed improvements in functional class (347 [15%] vs. 289 [12.6%]; *P* = not reported due to hierarchical analysis plan). Hypotension with systolic blood pressure of less than 100 mm Hg and elevations in serum creatinine of at least 2.0 mg/dL occurred less frequently in the sacubitril-valsartan arm, whereas angioedema occurred more frequently in the sacubitril-valsartan arm (14 [0.6%] vs. 4 [0.2%], *P* = 0.02). The investigators concluded that sacubitril-valsartan did not reduce the rate of a composite outcome of total heart failure hospitalizations and cardiovascular deaths compared with valsartan in patients with heart failure and preserved ejection fraction. Furthermore, any potential beneficial treatment effect appeared restricted to heart failure hospitalization and not cardiovascular mortality. The use of valsartan as an active comparator was consistent with most participants’ pre-enrollment treatment regimens but may have diminished any potential between-group differences.

In pre-specified subgroup analyses, significant heterogeneity of effect was observed by qualifying LVEF and gender. Sacubitril-valsartan reduced the primary composite outcome in patients with an ejection fraction below the median of 57% (rate ratio 0.78, 95% CI 0.64 to 0.95) but not those with an ejection fraction above the median of 57% (rate ratio 1.00, 95% CI 0.81 to 1.23) and among women (rate ratio 0.73, 95% CI 0.59 to 0.90) but not men (rate ratio 1.03, 95% CI 0.85 to 1.25). Although the results of pre-specified subgroup analyses in an overall neutral trial must be considered with caution, the intriguing interactions by LVEF and gender may prove to be significant for several reasons. Patients who have a preserved but modestly depressed ejection fraction may be phenotypically more similar to those with a markedly reduced than robustly preserved ejection fraction ^4^. Sacubitril-valsartan has proven benefits in patients with an ejection fraction of less than 40% ^5^. Moreover, post-hoc analyses of spironolactone ^6^ and candesartan ^7^ clinical trials suggest a benefit for these therapies in patients with a mid-range ejection fraction. The effect modification of gender merits further research. Although this unexpected finding may be attributable to chance, the known biological differences between men and women provide multiple potential explanations for future investigation. In addressing one question, PARAGON-HF has not only advanced our understanding of heart failure with preserved ejection fraction but also highlighted several areas for future research. It will be interesting to see whether guidelines incorporate the results of PARAGON-HF in a manner consistent with TOPCAT, as American guidelines give spironolactone a IIb recommendation for decreasing hospitalizations ^8^.

## Treatment of secondary mitral regurgitation

Although secondary mitral regurgitation due to left ventricular dysfunction confers an increased risk of hospitalization and death, treatment options remain limited as surgical repair or replacement does not improve clinical outcomes ^9, 10^. The MitraClip is a percutaneous device that reduces mitral regurgitation severity by facilitating approximation of the anterior and posterior mitral valve leaflets ^11^. The efficacy and safety of mitral valve repair with the MitraClip were studied in two clinical trials with similar designs.

MITRA-FR (N = 304) and COAPT (N = 614) were randomized comparisons of mitral valve repair with the MitraClip plus guideline-directed medical therapy versus guideline-directed medical therapy alone ^12, 13^. Although both trials enrolled patients with at least moderate to severe regurgitation, COAPT targeted a population of patients with a larger effective orifice regurgitant area (≥30 vs. ≥20 mm ^2^) or a larger regurgitant volume (>45 vs. >30 mL) and less severe left ventricular dysfunction (LVEF 20 to 50% vs. 15 to 40% and left ventricular end-systolic diameter of not more than 70 mL in COAPT vs. no restriction in MITRA-FR). Thus, the contribution of mitral regurgitation to heart failure symptoms relative to myocardial dysfunction may have been greater in COAPT than MITRA-FR. In addition, COAPT patients were enrolled after optimization of guideline-directed medical therapy.

In MITRA-FR, mitral valve repair with the MitraClip had no significant effect on the primary endpoint of all-cause death or heart failure hospitalization at 12 months (54.6% vs. 51.3% for repair vs. usual care; HR 1.16, 95% CI 0.73 to 1.83; *P* = 0.53). In contrast, in the COAPT trial, mitral valve repair with the MitraClip significantly reduced the risk of the primary endpoint of heart failure hospitalization at 24 months (35.8% vs. 67.9%; HR 0.53, 95% CI 0.40 to 0.70; *P* <0.001) and the composite of all-cause death or heart failure hospitalization ( *P* <0.001).

Pharmacologic treatment with sacubitril-valsartan may also reduce mitral valve regurgitation, although the effects on clinical outcomes remain unclear. In the PRIME trial, patients with heart failure and mitral valve regurgitation who were randomly assigned to sacubitril-valsartan had significantly greater reductions in effective orifice regurgitant area (−0.06 ± 0.10 vs. −0.02 ± 0.10 cm ^2^; *P* = 0.03) and regurgitant volume (−4.3 ± 15.1 vs. −11.6 ± 14.4 mL; *P* = 0.009) than those randomly assigned to valsartan. One (2%) death and three (5%) heart failure events occurred in the sacubitril-valsartan group versus zero and five (9%) in the valsartan group ( *P* >0.49 for each).

## SGLT-2 inhibitors and heart failure

The risk of incident heart failure in patients with type 2 diabetes mellitus (T2DM) is twofold greater than that of patients without T2DM ^14^. Moreover, the presence of T2DM is associated with a poor prognosis among patients with heart failure ^14^.

About 90% of tubular glucose reabsorption occurs through the tubular sodium-glucose co-transporter-2 (SGLT-2) ^15^. SGLT-2 inhibitors therefore lower blood glucose concentrations by enhancing glucosuria. SGLT-2 inhibition also induces durable weight loss (primarily a reduction in fat mass and not in lean mass) and lowers blood pressure ^16^.

Secondary and post-hoc analyses of three clinical trials provide strong evidence that SGLT-2 inhibitors modulate heart failure outcomes in patients with T2DM. Three SGLT-2 inhibitors (empagliflozin, canagliflozin, and dapagliflozin) significantly reduce the composite of heart failure hospitalization or cardiovascular death ^17– 19^. Whereas the beneficial effects of empagliflozin were consistent between patients with and without a history of heart failure at baseline ^20^, canagliflozin and dapagliflozin demonstrated larger effect sizes in patients with a history of heart failure ^21– 23^.

Small observational studies suggest that treatment of T2DM (with or without heart failure) with SGLT-2 inhibitors improves left ventricular filling pressure, as measured by E/e′ ratio and left atrial volume index ^24, 25^. The molecular mechanisms through which SGLT-2 inhibition may modulate cardiac structure and function remain under investigation. Hypothesized mechanisms include altered myocardial metabolism and energetics, glucosuria-induced diuresis without concomitant renin–angiotensin–aldosterone system activation, and inhibition of the myocardial sodium-hydrogen transporter ^10^.

Most recently, the DAPA-HF trial extended the benefits of SGLT-2 inhibitors from patients with T2DM to heart failure with reduced LVEF patients without T2DM ^26^. This trial randomly assigned 4744 patients with symptomatic heart failure and an ejection fraction of less than 40% to receive dapagliflozin 10 mg once daily or placebo, in addition to background heart failure therapy. The primary endpoint was hospitalization or urgent visit for heart failure or cardiovascular death. Most patients (58%) did not have a history of diabetes mellitus at baseline. The mean LVEF and the median NT-proBNP level at baseline were approximately 31 ± 7% and 1400 (857 to 2650) pg/mL, respectively. Use of guideline-directed medical therapy was high, including angiotensin-converting enzyme inhibitor (56%), angiotensin receptor blocker (27%) or sacubitril-valsartan (11%), a beta-blocker (96%), and mineralocorticoid receptor antagonist (71%).

After a median follow-up duration of 18.2 months, patients randomly assigned to dapagliflozin experienced significantly fewer primary composite events than those randomly assigned to placebo (16.3% vs. 21.2%; HR 0.74, 95% CI 0.65 to 0.85; *P* <0.001). Dapagliflozin also reduced the incidence of each of the individual components of the primary outcome. Furthermore, the benefits of dapagliflozin did not significantly differ between patients with (HR 0.75, 95% CI 0.63 to 0.90) and without (HR 0.73, 95% CI 0.60 to 0.88) T2DM at baseline ( *P* interaction = not reported). As expected, hypoglycemia requiring intervention occurred infrequently (4/2368 [0.2%]) in the dapagliflozin arm. There were no significant differences in amputations, diabetic ketoacidosis, or renal adverse events. These exciting results have re-positioned SGLT-2 inhibitors as a complete cardiometabolic, rather than glucose-lowering, therapy.

## Transthyretin amyloid cardiomyopathy

Aggregation of misfolded transthyretin monomers into amyloid fibrils and subsequent tissue deposition lead to tissue dysfunction ^27^. Myocardial infiltration of amyloid fibrils can cause heart failure by interfering with cardiac contractility and relaxation as well as through direct toxicity of the amyloid fibrils. Transthyretin amyloid cardiomyopathy may represent up to 12% of all cases of heart failure with preserved ejection fraction ^28^. Until the development of transthyretin stabilizers and RNA therapeutics, transthyretin amyloid cardiomyopathy treatment focused on symptom palliation and there was minimal impetus to diagnose this debilitating condition.

### Tafamidis

Tafamidis is a synthetic small molecule that binds to the thyroxine-binding sites on transthyretin ^29^. In adults with either wild-type or hereditary transthyretin amyloid cardiomyopathy, tafamidis significantly reduced the risk of all-cause mortality and cardiovascular hospitalization in the randomized, double-blind, placebo-controlled ATTR-ACT study ^30^. Of the 441 patients of ATTR-ACT, 71% (n = 186) of tafamidis patients were living compared with 57% (n = 101) of placebo patients at 32 months (win ratio 1.70, 95% CI 1.26 to 2.29; *P* <0.001). Cardiovascular hospitalization occurred in 138 tafamidis patients (52%; 0.48 per patient-year) compared with 107 placebo patients (61%; 0.70 per patient-year) (relative risk ratio 0.68, 95% CI 0.56 to 0.81; *P* = not reported).

The effects of tafamidis were consistent across transthyretin genotypes (for those with hereditary transthyretin amyloid cardiomyopathy) and both the 80 mg and 20 mg tafamidis doses. Delayed worsening heart failure symptoms and declining exercise capacity were observed in the tafamidis arms as early as 6 months, whereas the mortality benefit emerged after 18 months. ATTR-ACT was not powered to detect statistically significant improvements in left ventricular structure and function, although favorable trends were observed in left ventricular wall thickness and left ventricular global longitudinal strain. The incidence of serious adverse events was not significantly different between tafamidis and placebo.

### Patisiran

Patisiran is a small, interfering RNA ^31^ encapsulated within a liposome that targets a conserved sequence in the 3′ untranslated region of wild-type and mutant transthyretin mRNA, thereby suppressing gene expression via the RNA-induced silencing complex ^32^. Patisiran 0.3 mg/kg every 3 weeks decreased serum transthyretin levels by 81% and improved neuropathy, as measured by the modified Neuropathy Impairment Score + 7 in 225 patients with hereditary transthyretin amyloidosis enrolled in the randomized, double-blind, placebo-controlled APOLLO study (N = 225) ^33^.

Transthoracic two-dimensional echocardiography was performed in 126 (56%) APOLLO patients with left ventricular wall thickness of at least 13 mm and no history of aortic valve disease or hypertension ^34, 35^. Mean left ventricular wall thickness (least squares mean difference [LSMD] ± standard error of the mean [SEM], −0.9 ± 0.4; *P* = 0.017) and left ventricular end-diastolic volume (LSEM ± SEM, −5.1 ± 1.9 vs. −13.4 ± 3.4; *P* = 0.036) each decreased to a greater extent in the patisiran arm compared with placebo at 18 months. Patisiran improved left ventricular absolute global longitudinal strain by 1.4% (95% CI 0.3 to 0.5%; *P* = 0.02) versus placebo. Absolute basal, midwall and apical longitudinal strains also improved with patisiran treatment with basal longitudinal strain reaching statistical significance. In an exploratory post-hoc analysis of clinical outcomes, patisiran was associated with a trend toward lower risk of cardiac death or hospitalization compared with placebo (10.1 vs. 18.7 events per 100 patient-years; HR 0.54, 95% CI 0.28 to 1.01).

### Clinical implementation

Tafamidis was approved by the US Food and Drug Administration for the treatment of wild-type or hereditary transthyretin-mediated amyloid cardiomyopathy in 2019, whereas patisiran was approved for the treatment of hereditary transthyretin amyloid polyneuropathy, but not amyloid cardiomyopathy, in 2018. Both agents have a considerable cost of tens or hundreds of thousands of dollars annually. Thus, patisiran is unlikely to be covered by payers for the treatment of amyloid cardiomyopathy and tafamidis is unlikely to be covered for the treatment of polyneuropathy. Tafamidis is the preferred agent for patients with amyloid cardiomyopathy.

The effects of a second RNA therapeutic, inotersen ^36^, and a second transthyretin tetramer stabilizer, AG10 ^37^, on cardiac structure and function in adults with transthyretin amyloid cardiomyopathy are unclear.

## Iron deficiency

Depending upon the definition, iron deficiency (with or without anemia) affects up to 50% of adults with chronic heart failure ^38– 40^ and is associated with poor prognosis ^41^, impaired exercise capacity and skeletal muscle function ^42, 43^, and worse quality of life ^44^. Patients with heart failure have decreased myocardial iron content ^45^, and iron-deficient cardiomyocytes have impaired contractility ^46^ and mitochondrial dysfunction ^47, 48^. Iron repletion with intravenous iron improves quality of life and may prevent heart failure hospitalizations in patients with heart failure and iron deficiency, irrespective of anemia status ^49, 50^. In 2017, three clinical trials addressed important unanswered questions related to iron repletion in adults with heart failure, namely the effect of iron repletion on exercise capacity as measured by peak oxygen consumption ^50^, the effect of iron repletion on peripheral skeletal muscle function ^51^, and the role of oral iron supplements in patients with heart failure and iron deficiency ^52^.

### Effect of ferric carboxymaltose on exercise capacity in patients with iron deficiency and chronic heart failure (EFFECT-HF)

EFFECT-HF randomly assigned 172 adults with symptomatic heart failure with reduced ejection fraction and iron deficiency (defined as serum ferritin of less than 100 ng/mL or serum ferritin 100 to 300 ng/mL with a transferrin saturation of less than 20%) to receive ferric carboxymaltose, dose-adjusted to target hemoglobin, ferritin, and transferrin saturation levels, or usual care for 24 weeks ^49^. At 24 weeks, peak oxygen consumption decreased to a greater extent in the usual care arm than the ferric carboxymaltose arm (LSMD ± SEM, 1.0 ± 0.4 mL **·**kg ^−1^ **·**min ^−1^; *P* = 0.02). There were no between-group differences in ventilatory efficiency, as measured by the slope of the carbon dioxide–minute ventilation relationship, or treatment effect differences between patients with and without concomitant anemia.

Thus, EFFECT-HF was the first trial to demonstrate an improvement in exercise capacity using gas-exchange variables rather than the 6-minute walk test. In their joint 2017 focused update of the Guideline for the Management of Heart Failure, the American College of Cardiology Foundation and the American Heart Association gave intravenous iron replacement to improve function status and quality of life a weak recommendation (IIb) based upon moderate-quality evidence (B-R) ^8^. EFFECT-HF was not included in the focused guideline update.

### Ferric iron in heart failure II (FERRIC-HF II)

FERRIC-HF was a randomized, double-blind, placebo-controlled clinical trial of 40 patients with symptomatic heart failure with reduced ejection fraction, iron deficiency (defined as serum ferritin of less than 100 ng/mL or serum ferritin 100 to 300 ng/mL with a transferrin saturation of less than 20%), and normal folate and vitamin B _12_ levels ^51^. Patients randomly received iron isomaltoside (the total dose was calculated by using the Ganzoni formula) or matching placebo. The primary endpoint was phosphocreatine recovery half-time on dynamic 31P magnetic resonance spectroscopy during submaximal exercise, where a shorter half-life indicates faster phosphocreatine recovery and improved mitochondrial oxidative function.

At 2 weeks, phosphocreatine half-time was −6.8 seconds (95% CI −11.5 to −2.1; *P* = 0.006) shorter in the iron isomaltoside group than the placebo group. Iron isomaltoside also improved adenosine diphosphate recovery half-time but had no effect on resting or end-exercise phosphocreatine or adenosine diphosphate half-time. This study provides important mechanistic insight into the pleiotropic effects of iron repletion in heart failure.

### Iron repletion effects on oxygen uptake in heart failure (IRONOUT HF)

**Figure 2.**
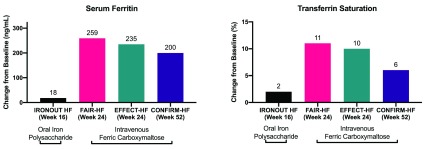
Comparison of iron repletion regimens in patients with heart failure.

In IRONOUT HF, 225 patients with symptomatic heart failure with reduced ejection fraction and iron deficiency (defined as serum ferritin of less than 100 ng/mL or serum ferritin 100 to 300 ng/mL with a transferrin saturation of less than 20%) were randomly assigned to receive iron polysaccharide 150 mg twice daily for 16 weeks ^52^. Oral iron supplementation with iron polysaccharide had no effect on peak oxygen consumption, ventilatory efficiency, 6-minute walk distance, or heart failure symptoms. Notably, iron polysaccharide had minimal effects on serum ferritin (median change from baseline of 18 ng/mL, 95% CI −8 to 38) and transferrin saturation (median change from baseline of 2%, 95% CI −3 to 7%). Figure 2 compares changes in serum ferritin and transferrin saturation between oral and intravenous iron repletion regimens in patients with heart failure at multiple time points. Subgroup analyses suggest that iron repletion was greater among patients with lower levels of hepcidin, an iron regulatory protein that decreases enteral iron absorption and sequesters iron intracellularly.

Future studies should determine whether different oral iron regimens can replete iron stores in patients with heart failure. Studies of women with anemia suggest that frequent oral iron administration induces an increase in hepcidin levels and that less frequent oral supplementation (for example, once daily on three days of the week) paradoxically may improve iron repletion ^52, 53^.

## Biomarker-guided heart failure therapy

The natriuretic peptide B-type natriuretic peptide (BNP) and its congener N-terminal-proBNP (NT-proBNP) provide considerable diagnostic and prognostic value in heart failure ^19^. Yet the value of natriuretic peptide–guided heart failure management remains unclear. A meta-analysis of 2000 participants across 11 clinical trials demonstrated significant reductions in all-cause mortality (HR 0.62, 95% CI 0.45 to 0.86; *P* = 0.004), heart failure hospitalization (HR 0.80, 95% CI 0.67 to 0.94; *P* = 0.009), and cardiovascular hospitalization (HR 0.82, 95% CI 0.67 to 0.99; *P* = 0.048) with natriuretic peptide–guided heart failure management ^54^. In contrast, the GUIDE-IT trial (N = 894) found no difference in time to first heart failure hospitalization or cardiovascular death between patients randomly assigned to an NT-proBNP–guided strategy and usual care ^55^.

An exploratory, post-hoc analysis of the neutral TIME-CHF trial used the gap-time method to compare NT-proBNP–guided therapy with usual care to account for recurrent events ^56^. While NT-proBNP–guided therapy was associated with reduced second all-cause hospitalizations, there was no effect on the second heart failure hospitalization. In the subgroup of patients younger than 75 years, guided therapy was associated with reduced first and second all-cause and heart failure hospitalizations. Current guidelines do not recommend natriuretic peptide–guided therapy ^57, 58^.

Carbohydrate antigen 125 is a glycoprotein associated with prognosis in acute heart failure. In a multicenter clinical trial of 380 patients, carbohydrate antigen 125–guided therapy significantly reduced the composite of death or heart failure hospitalization at 1 year. Replication of these results would provide compelling support for carbohydrate antigen 125–guided therapy.

## Remote hemodynamic monitoring-guided heart failure therapy

Titration of guideline-recommended medical therapy to a target pulmonary artery diastolic pressure, estimated using an implantable hemodynamic monitor, reduces heart failure hospitalizations by 28% in patients with reduced and preserved ejection fraction ^59, 60^. Recent analyses have provided additional insights into the efficacy and safety of remote hemodynamic monitoring. First, the findings of the CHAMPION trial have been replicated in two separate analysis of routinely collected clinical data ^61, 62^. Second, review of medication titration patterns during the CHAMPION trial has demonstrated that diuretic adjustments and (among patients with heart failure and a reduced ejection fraction) guideline-directed medical therapy adjustments contributed to the reduced hospitalization rates ^63, 64^. Third, individual practices have begun to report their experience with the implementation and maintenance of a remote hemodynamic monitoring program ^65– 67^. These practice-based insights will prove useful as additional remote monitoring devices reach the market. Last, comparative effectiveness studies suggest that remote hemodynamic monitoring meets currently accepted thresholds for cost-effectiveness but the overall budget impact may be difficult to absorb ^68, 69^. Ongoing research is investigating the role of remote hemodynamic monitoring in patients with mechanical circulatory support devices and the effects of novel heart failure therapies on pulmonary artery pressure ^70– 72^.

## Conclusions

After more than a decade of relatively modest advancements, heart failure therapeutic development has accelerated and led to several advances in the treatment of chronic heart failure. Some of these new technologies improved clinical outcomes, whereas others improve functional or patient-reported outcomes.
